# Food and healthcare accessibility during COVID-19 pandemic

**DOI:** 10.1016/j.heliyon.2021.e08656

**Published:** 2021-12-22

**Authors:** Emmanuel Uche, Samuel Nnamdi Marcus, Lionel Effiom, Chijioke Okoronkwo

**Affiliations:** a Department of Economics, Abia State University, Uturu, Abia State, Nigeria; b Department of Economics, University of Calabar, Calabar, Cross River State, Nigeria; c Central Bank of Nigeria, Nigeria

**Keywords:** COVID-19, Food prices, Healthcare prices, Government stringency measure, Containment health index, Dynamic ARDL

## Abstract

The pervasive effects of the novel coronavirus (COVID-19) have put the world to test. Its effects permeate all facets of life including healthcare services and food supplies. However, most empirical studies failed to investigate its effects on the prices of food and healthcare services, which by all standards, are essential commodities. On this background, this study evaluates the impact of COVID-19 reported cases and lockdown stringency measures on the food and healthcare prices in the six (6) worst-affected countries. For empirical purposes, daily prices of food and healthcare services between 22^nd^ January and 31^st^ December 2020 were regressed against daily cases of COVID-19 and lockdown stringency measures within the dynamic autoregressive distributed lag procedure. Empirical evidences reveal that prices of healthcare and food are cointegrated with COVID-19 cases and lockdown measures in all the selected countries except Italy. Equally, healthcare and food prices reinforced itself in the long-run in the US, the UK and France. Furthermore, COVID-19 cases lead to significant increases in food and healthcare prices in the US, whereas, food and healthcare prices in France and UK declined significantly as COVID-19 cases mount. Conversely, food and healthcare prices declined significantly in the US and soar in France and the UK in reactions to COVID-19 new cases. Likewise, government stringency measures and containment health measures contributed significantly to healthcare and food price hike in the US and France respectively. Meanwhile, healthcare and food prices in the other selected countries remained unaffected even as the pandemic ravages. Following this empirical discoveries, relevant policy guidelines have been communicated.

## 1 Introduction

Beginning from late December 2019, the world has been battling with a common enemy known as corona virus (COVID-19). As exposed in several studies, including (Ashraf, 2020; Tandra et al., 2021) among others, the virus was first recorded in Wuhan region of China in December 2020. Meanwhile, on the 11^th^ day of March 2020, due its wide spread across all regions of the globe, the World Health Organization (WHO) officially announced that the virus has acquired the status of a global pandemic (Zhang et al., 2020). Consequently, the increasing cases of the virus and subsequent death cases in most part of the world led governments to impose stringent measures to contain the spread. Such measures include restrictions in the movements of persons, goods and services, and subsequent closure of several business outlets with its severe economic implications. It also had direct or indirect negative impact on several aspects of the economy including food supply and healthcare service delivery among other related issues. Intuitively, the rising cases of COVID-19 and the lockdown measures may have impacted greatly on the prices of food items and healthcare services mostly in the worst affected countries. In this light, Agyei et al. (2021) suggest that equilibrium price changes as a result of changes in supply and demand for food during the pandemic. Also, the restrictions introduced in the wake of the COVID-19 outbreak have battered both the supply and demand sides of the food market. Demand and supply forces in the food market are influenced by both COVID-19 cases and the consequential lockdowns instituted as measures to curtail the infection rate in the absence of a vaccine. Such effects could apply to healthcare prices mainly due to rising cases and the subsequent lockdown measures even in countries with robust healthcare programs. However, scholars have, to a large extent, remained silent about such pass-through effects leading to the current investigation.

In the worst-affected countries, including the US, Turkey, Italy, the UK, Russia and France, the recorded cases of the novel virus exceeded 2 million as at 31^st^ December, 2020^1^. Specifically, the US, France, Russia, the UK, Turkey and Italy recorded above 2 million cases as at June 10^th^, November 14^th^, November 20^th^, December 19^th^, December 19^th^ and December 24^th^ respectively. The number of covid-19 reported cases soared to 20,000,595 (345,955 death cases) in the US as at 31^st^ December, 2020 making her the most affected among others. Coming behind the US in terms of COVID-19 reported cases as at 31^st^ December, 2020 is Russia (3,127,347), France (2,677,666), the UK (2,496,231), Italy (2,009,317) and Turkey (2,004,285). The reported cases of transmissions and deaths continued rising amid varying degrees of lockdown policies including government stringency measure and containment and health measures adopted by individual countries.

As earlier highlighted, available evidences eloquently revealed that previous studies, except Agyei et al. (2021), failed to examine the link between COVID-19 cases and lockdown stringency measures on the prices of food and healthcare services. Rather, they are inclined to investigating the effects of COVID-19 on several other factors, such as stock market returns and volatility (Narayan et al., 2020; Salisu et al., 2020; Ashraf, 2020; Zoungrana et al., 2021; Sharma et al., 2021), financial markets (Zhang et al., 2020), monetary policy transmissions (Wei and Han 2021), oil prices (Devpura and Narayan, 2020; Narayan, 2020; Zhang and Hamori, 2021), economic growth (Jena et al., 2021), exchange rates (Narayan et al., 2020), and housing prices (Qian et al., 2021), firm performance (Hu and Zhang, 2021), cryptocurrency (Sahoo, 2021). Consequently, the unavailability of empirical studies investigating the potential influence of COVID-19 reported cases, government stringency measures and containment and health measures on consumers’ access to food supply and healthcare services eminently incentivizes the current enquiry.

In the light of this backdrop, the objective of the present study is to critically evaluate empirically, the effects of COVID-19 reported cases and lockdown stringency measures (Government Stringency Index and Containment and Health Index)^2^on food and healthcare prices in the six worst-affected countries. We hypothesize, therefore, that rising cases of COVID-19 and lockdown stringency measures could impede consumers access to food supply and healthcare services albeit rising prices. We equally envisaged that the restricted access to food items and health care services could further aggravate the mortality rate, thereby casting doubt on the ability of government to contain the spread. This is so expected, as O'Hara and Toussaint (2021) highlighted that the inability of the populace to have unhindered access to the necessary food supplies and healthcare services arising from price instability mainly due to restrictions in movements could make them more vulnerable to the attack of the virus. To this end, it is expected that the findings of the current study will draw government's attention to the potential effects of COVID-19 cases and its induced lockdown stringency measures on the prices of food and healthcare services. Meanwhile, a welfare-centric government is expected to ensure that the hardship occasioned by the virus and subsequent lockdown stringency measures are drastically minimized. Consequently, appropriate policy guidelines will be communicated leading to welfare maximizations. Also, appropriate policy guideline to withstand and shield the economy (food and healthcare prices) from such outbreak in the future will be elicited. Based on the aforementioned, the following research question is germane: to what extent does the rising cases of COVID-19 impacted on food and healthcare prices in this six (6) worst-affected countries? How do food and healthcare prices respond to lockdown stringency measures?

On account of its guiding objectives and mindful of extending the trajectory of existing knowledge, the current study differs from existing literature in several respects. First, unlike previous studies, the current study considers the relative effects of COVID-19 and its induced lockdown stringency measures on both food and healthcare prices in the selected worst-affected countries. Secondly, as against panel aggregated study which may suffer aggregation bias, the current study provides country specific estimations leading to country unique policy directions devoid of aggregation bias. Equally, the inclusion of the containment and health index in the model makes the study unique noting that previous studies did not consider its effects on macroeconomic variables. Moreso, the application of the recently developed Dynamic Autoregressive Distributed Lag (DARDL) model makes the current study exceptional considering its unique capacity in revealing the simulative counterfactual effects of the explanatory variables on the explained variable (Ali et al., 2021; Amin et al., 2021). To the best of our knowledge, no study in the past took advantage of this enhanced econometric technique in the analysis of COVID-19 related studies. Therefore, profound and evidence-based policy guidelines that will enhance the welfare of the populace and strengthens governments’ intervention policies in terms of access to food supply and healthcare services are expected from this study.

The remaining part of the study is concisely organized thus: literature review is presented in part-2, followed by methodology presented in part-3. The empirical analysis and discussions of findings are outlined in part-4, while the study is summarized with some policy recommendations in part-5.

## 2 Review of related literature

The review is focused on the Grossman's theory of demand for healthcare developed in 1972. Grossman (as cited in Muurinen, 1982) argues that the concepts of health is a durable capital good which is inherited but depreciates over time, and investment in health as an activity where medical care is combined with other inputs in order to produce new health so as to partly counteract the gradual natural deterioration of health. Consequent to the framework of the household production theory, the Grossman model posits that health is both a consumption and an investment commodity, implying that individuals are both consumers and producers of health. As a producer, the individual invests to produce health by obtaining inputs such as medical care, diet and clothing, exercise etc. to produce health. In the model, the health of an individual is treated as a durable and endogenously determined capital stock (Hren, 2012). That is, the length of an individual's health is endogenously determined since health depends on the amount of resources allocated to the production of health.

Although the Grossman model has severely been criticized since it was conceived, it still remains a unique approach within health economics to treat both empirically and theoretical issues on demand for health. For instance, on the issue of assumption of certainty in Grossman model, Dowie (1975) argues that the basic model is unduly unrealistic considering the inherently uncertain area of health and utilization behavior. Muurinen (1982) criticized the credibility of the dichotomy of health stock in Grossman's model into direct increase in utility which is consumption benefits and increased health time available for activities which is investment benefits. He argued that treating health benefits as alternative specifications or hypotheses seems to be intuitively wrong since health is demanded for both utility consequences-that is relief of pain and for functional capacity consequences-better performance of necessary tasks. Though Muurinen (1982) retained the separation of health benefits into two, he rather treated them not as alternatives but as explicitly complementary since both are produced from the same addition to the stock of health.

Recently, Khan et al. (2021) identified a link between COVID-19 and the macroeconomy by categorizing the costs into tangibles and intangibles. Accordingly, the tangible costs include; increases in unemployment, inflation, decreases in food supply, productivities, investments, tourism receipts, etc. Likewise, the intangible costs comprise of reduced standard of living, increased emotional trauma, etc. Building upon this background, the current study traces the immediate impact of COVID-19 on inflation, a tangible cost proxy by daily prices of food and healthcare in the 6 worst affected countries.

In this present study, we posit that the stock of health assumed to be endogenously determined is unsatisfactory. Our study argues that in certain situations when the stock of an individual's health is exogenously dependent on social and public-governmental factors, like the COVID-19 situation where individual's resources could not increase the stock of wealth but by complying with some stringent measures from medical practitioners and restrictions imposed by the government. We therefore submit that an individual's stock of health is influenced by both endogenous and exogenous factors. COVID-19 therefore could influence the demand and supply for health, which will likely affect its prices amid healthcare plans and insurance in some countries. First, is the increase in the demand for healthcare resulting from the pandemic even in the face of low income, as a good number of people were affected. Second is the decrease in supply of healthcare resulting from the stringent measures from regulatory agencies and restrictive measures from the government. These measures include restriction of movement, social distancing to mention but a few which to a great extent determined the quantity and quality of health supplied. Similarly, the marked influence of COVID-19 on food prices is a technical function of the lockdown measures which inhibited free and smooth movement of people and food items. The resultant effect of these measures includes scarcity and high cost of transportation which hikes the prices of food.

Likewise, several other studies have attempted establishing a link between COVID-19 and macroeconomic fundamentals. Meanwhile, the ensuing debate cut across several global economies and various macroeconomic factors. Accordingly, we have outlined the most relevant studies to shed more light on the dynamics and to buttress our arguments with respect to the focus of the current study. First, Agyei et al. (2021) estimated COVID-19 and food prices in Sub- Sahara Africa with controls for macroeconomic setting using general method of moments estimation. The study found that the COVID-19 outbreak led to increases in food prices of the sampled countries. Restrictions on movements or lockdowns in the wake of COVID-19 were associated with an increase in the price of maize only. The study further found that exchange rate, inflation and crude oil prices exerted a detrimental effect on food prices. Although Agyei et al. (2021) considered the effects of COVID-19 on food prices, but the study did not factor in the effects of the pandemic and the lockdown stringency measures on healthcare prices. The study also enlisted only sub-Saharan African countries while ignoring the worst-affected countries. Likewise, Sharma et al. (2021) examined the relationship between COVID-19 cases, temperature, exchange rate and stock returns in the top-15 most affected countries through the application of wavelet and wavelet partial coherences procedures. The study confirms that COVID-19 cases have a long-term effect on exchange rate and stock returns in all the selected countries. Closely related to the above study is Goswami et al. (2021) which sought to explain the impact of COVID-19 on economic performances of the states of India using panel regression analysis. The study affirms that states with higher incidences of COVID-19 suffered more economic losses relatively to the ones with fewer cases. This demonstrates the negative influence of COVID-19 on the economy, however, the study failed to consider specifically, how the prices of food and healthcare services were affected by the pandemic.

A similar study is Hu et al. (2021) that considered the influence of COVID-19 cases on housing prices in Australian states using daily hedonic returns. The investigation reveals that COVID-19 cases exert negative influence on housing prices. The study further confirms that COVID-19 lockdown measures have insignificant effects on housing prices. However, the study failed to use a definitive measure of lockdown measure like the stringency and health containment measure which makes the current study so relevant. Qian et al. (2021) also considered the impact of COVID-19 on housing prices in China using the difference-in-difference technique. Accordingly, the study reports that housing prices in communities with reported cases would decline to the tune of 2.4 per cent as COVID-19 cases increases. Based on SVAR method, Lee et al. (2021) investigated the impact of COVID-19 pandemic and macroeconomic fluctuations on hospitality stock returns in China. From the evidence recorded, the study specifically states that an unanticipated positive change in COVID-19 cases leads to significant negative changes in hospitality stock returns.

In another dimension, Salisu et al. (2020) evaluated the predictive power of global fear index (GFI) during COVID-19 on commodity prices within the globe. Through the application of in-sample and out-of-sample techniques, the study confirms that commodity price rises as COVID-19 related fear rises. Although the study is closely related to the current investigation, however, it failed to point out the specific commodities with such rising prices. In a related study, Anser et al. (2021) examined the role of COVID-19 testing and functional laboratories on financial development within a panel of 115 countries. The study specifically confirms that financial market was disrupted due to COVID-19 recorded cases. Likewise, a review of empirical papers regarding economic crises by Alam et al. (2020) concludes that COVID-19 had significant impact on different sectors of Bangladesh economy including the food and agriculture, ready-made garments, bank and financial institutions, foreign remittances, etc. Meanwhile, Shafi et al. (2020) report that COVID-19 pandemic had adverse effects on the performances of micro, small and medium-size enterprises (MSMEs) in Pakistan. Additionally, Devpura and Narayan (2020) used different measures of oil price volatility to access the evolution of oil prices during the pandemic. After controlling for conventional predictors of oil price volatility, they submit that covid-19 cases and deaths contributed significantly to daily oil price volatility. Feng et al. (2021) applied GMM technique in a panel study of 20 countries to estimate the effects of COVID-19 cases and government interventions on exchange rate volatility during the pandemic. The study reports that rising cases of COVID-19 leads to increasing volatility of exchange rate whereas government interventions inhibit exchange rate volatility.

Going further, Huo and Qiu (2020) evaluated the response of China's stock market to the pronouncement of COVID-19 lockdown measures. They observed reversals at both industry and firm levels due to the pronouncements of containment measures. Related to the above is He et al. (2020) that investigated the impact of COVID-19 on stock market in 8 countries including China, Italy, South Korea, France, Spain, Germany, Japan and the US. Based on the results obtained through the conventional *t*-tests and the non-parametric tests of Mann-Whitney, they confirm that COVID-19 has a short-term negative impacts on stock market in all the countries considered. However, the study did not provide any detail about the long-run effects. Jena et al. (2021) applied the multilayer artificial neural network forecaster in the study of COVID-19 impact on the GDP of major economies. The study confirms a negative reaction of the GDP to COVID-19 cases in all the selected economies. Reissl et al. (2021) equally assessed the economic implications of the COVID-19 and lockdown measures in Italy using a dynamic input-output measure. The study reports that labour supply was the most affected due to COVID-19 induced lockdown measures. Similarly, Wei and Han (2021) applied the event study technique in accessing the transmissions of monetary policy to the financial market during COVID-19 pandemic in 37 countries with severe cases. They suggest that COVID-19 pandemic significantly inhibited the transmissions of monetary policy to the financial markets. Also, Abu et al. (2021) through various estimation procedures, including ARDL, FMOLS highlight a significant negative and significant positive impacts of COVID-19 reported cases and deaths cases, respectively on stock market performances in Nigeria. Likewise, evidence from Zoungrana et al. (2021) and Nguyen et al. (2021) confirmed the negative effects of COVID-19 pandemic on stock listed on West African Economic and Monetary Union (WAEMU) and the US and China equity markets volatility spillover on global equity market volatility, respectively. More so, Narayan et al., 2020a, Narayan et al., 2020b highlight that COVID-19 cases and travel bans had positive effects on G7 stock markets.

Based on the above empirical expositions, a critical observation aptly demonstrates that most prior studies failed to consider the potential effects of COVID-19 pandemic and its induced lockdown stringent measures on food and healthcare prices in the worst-affected countries. Also, the effect of government stringency index and health containment index on food and healthcare prices were entirely missing. On this background, the current study is poised to fill this identified lacuna in literature with respect to studies that considered the impacts of COVID-19 pandemic on the macro-economy.

## 3 Data descriptions and model building

### 3.1 Data description

To provide empirical details, the study made use of daily frequency data ranging from 22^nd^ January to 31^st^ December, 2020 for the US, 24^th^ January to 31^st^ December, 2020 for France, 31^st^ January to 31^st^ December, 2020 for Italy, the UK and Russia federation. Finally, that of Turkey is between 11^th^ March and 31^st^ December, 2020. Accordingly, making total observations of 345, 343, 336 and 296 for the US, France, Italy, the UK, Russia and Turkey in that other. Meanwhile, the data sets on food and healthcare prices were originally monthly frequencies. The monthly series were subsequently converted into daily frequencies through the quadratic match-sum procedure based on the insights gained from Shahbaz et al. (2018) and Uche and Effiom (2021). Ideally, the quadratic match-sum process enables the conversion of low-frequency data sets into high-frequency series and it permits amendments for seasonal variations through dropping end-to-end data dispersions (Shahbaz et al., 2018). Meanwhile, Table 1 represents the summary of the data in terms of notations, unit of measurements and sources.

**Table 1:** Data description and sources.

Variable Notation	Series	Unit of measurement	Source
CVC	COVID-19 reported Cases	Daily recorded cases	Our World in Data. https://ourworldindata.org/coronavirus-data?country
CVNC	COVID-19 New Cases	New cases recorded on daily basis	Our World in Data. https://ourworldindata.org/coronavirus-data?country
GSI	Government Stringency Index	Composite policy indicator based on nine policy indicators rescaled to a value from 0 to 100 (100 = strictest).	Our World in Data. https://ourworldindata.org/coronavirus-data?country
CNHI	Containment and Health Index	Composite policy indicator based on thirteen policy indicators rescaled to a value from 0 to 100 (100 = strictest).	Our World in Data. https://ourworldindata.org/coronavirus-data?country
FDP	Food Prices	Consumer Price Index (CPI) for Food and non-alcoholic beverages	Country Harmonized Index and Weights - International Financial Statistics (IFS).
HCP	Healthcare Prices	Consumer Price Index (CPI) for Healthcare	Country Harmonized Index and Weights - International Financial Statistics (IFS).

**Note:** All the data sets are available and freely obtainable in public data repositories.

As clearly noted in^3^, government stringency index (GSI) and containment and health index (CNHI) vary between 0 and 100. The indices (GIS and CNHI) indicate only the extent of strictness with a score of 100 showing the highest strictness. Meanwhile, it is further highlighted that higher score does not necessarily imply that the policies are more effective or more appropriate than lower score. Further evidences on the nature and dynamics of the relevant data sets and for each selected country are succinctly provided in Table 2 accordingly.

**Table 2:** Summary statistics.

Variable	Mean	Std. Dev.	Skewness	Kurtosis	Jarque-Bera
**USA**					
FDP	3.82	0.08	-0.23	2.32	9.16∗∗
HLP	4.23	0.08	0.14	2.21	9.21∗∗
CVC	14.28	2.97	-2.46	8.86	765.7∗∗∗
CVnC	10.26	2.15	-2.97	12.67	1681∗∗∗
GSI	4.12	0.46	-4.07	19.28	4324∗∗∗
CnHI	4.07	0.38	-4.08	19.44	4399∗∗∗
**Italy**					
FDP	3.46	0.07	0.12	2.16	9.95∗∗∗
HLP	3.33	0.06	0.33	2.31	12.21∗∗∗
CVC	12.26	1.91	-2.83	14.26	2095∗∗∗
CVnC	7.48	1.82	-0.23	3.14	3.01
GSI	4.16	0.25	-0.58	4.63	52.97∗∗∗
CnHI	4.14	0.18	-1.08	8.17	415∗∗∗
**France**					
FDP	3.54	0.07	-0.01	1.99	12.93∗∗∗
HLP	3.16	0.06	0.35	2.41	10.80∗∗∗
CVC	12.11	2.64	-2.28	8.85	704∗∗∗
CVnC	7.64	2.28	-1.09	4.49	89.78∗∗∗
GSI	4.06	0.39	-2.04	9.27	715∗∗∗
CnHI	4.06	0.29	-2.93	14.51	2131∗∗∗
**The UK**					
FDP	3.41	0.07	0.44	3.73	18.05∗∗∗
HLP	3.69	0.08	0.05	2.56	2.74
CVC	11.86	2.88	-2.10	6.75	431∗∗∗
CVnC	7.59	2.32	-1.47	5.56	208∗∗∗
GSI	4.03	0.58	-2.33	6.70	482∗∗∗
CnHI	3.94	0.41	-2.31	6.79	485∗∗∗
**Russia**					
FDP	6.37	0.15	-0.36	2.51	9.82∗∗
HLP	6.53	0.16	-0.13	2.36	5.96∗
CVC	12.62	2.80	-2.24	7.62	520∗∗∗
CVnC	8.58	1.84	-2.57	10.06	960∗∗∗
GSI	4.05	0.33	-1.53	9.30	616∗∗∗
CnHI	4.06	0.26	-3.01	21.43	4717∗∗∗
**Turkey**					
FDP	6.28	0.23	0.63	2.43	23.78∗∗∗
HLP	6.13	0.17	0.16	2.13	10.35∗∗∗
CVC	12.05	1.92	-3.25	16.90	2875∗∗∗
CVnC	7.58	1.34	-1.16	11.44	938∗∗∗
GSI	4.14	0.19	-1.92	8.74	583∗∗∗
CnHI	4.11	0.13	-3.09	18.70	3478∗∗∗

Note: The Table provides a summary for all the variable in all the selected countries in terms of average, spread and normality. ∗∗∗, ∗∗, ∗ indicate that the variables are not normally distributed leading to the rejection of null hypothesis of normality at 1%, 5% and 10% levels of significance respectively. All variables are expressed in their logarithmic values except FDP and HLP that are in their original index value.

From the summary statistic in Table 2, it is observed that the USA has the highest COVID-19 expected value (14.28), followed by Russia (12.62), Italy (12.26), France (12.11), Turkey (12.05), and the UK (11.86). All the series deviate from normal curve except *CVnC* in Italy and HLP in the UK based on the probability values the Jarque-Bera statistics.

### 3.2 Model building

Our study is anchored on the Grossman's theory of demand for health. The appropriateness of this theory is viewed from the stance that it contains individual producer and consumer analyses, hence, can be transformed into producer and consumer models. In its general form, the dynamics of Health in Grossman's model is given as:

(1) H_t_ = l_t_ (M_t_, t^i^) – δ_t_ H_t_

where

H_t_ = Health capital

l_t_ = gross investment during the interval t

M_t_ = medical care

t^i^ = own time on sport for instance

δ = rate of health capital depreciation dependent on individual's age and exogenously determined Eq. (1) suggests that health is a function of gross investment produced by medical care and own time spent on sporting activities while health capital depreciate at an assumed constant rate. Our study transforms Eq. (1) by introducing some exogenous variables of interest to explain the influence on individual's health during the COVID-19 period. We therefore hypothesize that:

(2) HLP = *f* (CVC, CVNC, GSI, CNHI)

Similarly, we assume that food prices (FDP) a key component of the consumer price index during the COVID-19 period is affected by same exogenous factors in Eq. (1) thus:

(3) FDP = *f*(CVC, CVNC, GSI, CNHI)

Based on the aforementioned objectives of this research and following insights gained from Amin et al. (2021) the current study employed the Dynamic Autoregressive Distributed Lag (DARDL) model. Ideally, the DARDL introduced by Jordan and Philips (2018) is an enhanced version of standard ARDL model. Accordingly, the DARDL modeling procedure complies with all the standard requirements of the conventional ARDL process in terms of stationarity tests and bounds tests of long-run relationship (Amin et al., 2021). According to Jordan and Philips (2018), the DARDL model, much unlike the VAR based impulse response function (IRF), the DARDL simulations reveal more clearly the relative effects of the shocks from the predictor on the response variable. It simulates the counterfactual effects of the regresor on the regresand (Amin et al., 2021). Besides the estimation of short- and long-run effects, the DARDL model produces unique graphical illustrations, much like the VAR Impulse Response Function that demonstrate the response of the explained variable to innovations in the explanatory variable(s). Accordingly, the typical pathway of the DARDL framework is demonstrated below:

(4) $$\text{Δ}{!}_{kt}=\beta_{0}+\beta_{1}{\left(!\right)}_{kt-1}+\beta_{2}{\left(\text{X}\right)}_{kt-1}+\ldots +\beta_{3}{\left(\text{X}k\right)}_{t-1}+\overset{p}{\underset{i=1}{\sum }}\theta_{1}\text{Δ}{\left(!\right)}_{t-i}+\overset{q}{\underset{i=1}{\sum }}\theta_{2}\text{Δ}{\left(\text{X}k\right)}_{t-i}+\ldots +\overset{q}{\underset{i=1}{\sum }}\theta_{3}\text{Δ}{\left(\text{X}\right)}_{t-i}+{\text{ε}}_{t}$$

where Ɣ is the dependent variable, Δ is the lag operator showing short-term changes, $\beta_{0}$ is the constant coefficient, Ӽ_1_ and Ӽ_k_ are sets of independent variables, $\beta_{1},\beta_{2},and\beta_{3}$ are coefficients of long-term parameters, $\theta_{1},\theta_{2}and\theta_{3}$ are coefficients of short-run parameters, while ${\text{ε}}_{t}$ is the stochastic variable.

Following the DARDL pathway represented in Eq. (4), we provide forthwith the dynamic association between COVID-19 and its induced stringency measures on food and healthcare prices.

Model 1:

(5) $$\text{Δ}ln_{\text{HLP}}=\varphi_{0}+\beta_{0}lnHLP_{t-i}+\beta_{1}lnCVC_{t-i}+\theta_{1}\text{Δ}lnCVC_{t-i}+\beta_{2}lnCVNC_{t-i}+\theta_{2}\text{Δ}lnCVNC_{t-i}+\beta_{3}lnGSI_{t-i}+\theta_{3}\text{Δ}lnGSI_{t-i}+\beta_{4}lnCNHI_{t-i}+\theta_{4}\text{Δ}lnCNHI_{t-i}+{\text{ε}}_{t}$$

Model 2:

(6) $$\text{Δ}ln_{\text{FDP}}=\varphi_{0}+\beta_{0}lnFDP_{t-i}+\beta_{1}lnCVC_{t-i}+\theta_{1}\text{Δ}lnCVC_{t-i}+\beta_{2}lnCVNC_{t-i}+\theta_{2}\text{Δ}lnCVNC_{t-i}+\beta_{3}lnGSI_{t-i}+\theta_{3}\text{Δ}lnGSI_{t-i}+\beta_{4}lnCNHI_{t-i}+\theta_{4}\text{Δ}lnCNHI_{t-i}+{\text{ε}}_{t}$$

where all notations remain as previously defined, models 1 and 2 (Eqs. (5) and (6)) represent the dynamic effects of COVID-19 and its induced stringency measures on healthcare and food prices respectively in each of the six (6) worst affected countries. The implementation of the Dynamic ARDL (DARDL) of Eqs. (5) and (6) are based on 5000 simulations of the vector from the multivariate normal distributions following Amin et al. (2021).

## 4 Empirical results and discussions

Before the implementation of the DARDL procedure, two important pre-estimation requirements must be satisfied. First, all the variables entering the model must be stationary at most after first difference. That is, none of the variable is expected to be integrated of order-two *I(2)*. Second, a test of cointegration must be performed to ascertain whether the variables share a common long-run relationship. When a long-run relationship exists among the variables, the dynamic simulations of the variables henceforth becomes appropriate and applicable (Nwani and Omoke, 2020). Accordingly, the study proceeds with the test of stationarity using the standard augmented Dickey-Fuller and the Philips-Perron unit-root tests. The test of cointegration is based on the Pesaran et al. (2001) bounds test procedure. The estimated results of the unit-root tests and the cointegration test are summarized in Tables 3 and 4 respectively. Evidence arising from the test of stationarity shows that the data sets are mutually integrated. That is, some are integrated of order-zero *I(0)* while some are integrated of order-one *I(1)*. Accordingly, the evidence of mutual integration of the data sets cut across all the sample countries. Having confirmed the maximum order of integration among the series (order-one), moreso, that the series are mutually integrated between orders-zero and one, the study therefore, proceeds with the test of cointegration through the enablement of the PSS bounds test cointegration technique.

**Table 3:** Unit-tests result.

Series	Augmented Dickey-Fuller (ADF)			Philips-Perron (P–P)		
	ADF Levels *I(0)*	ADF 1^st^ Diff. *I(1)*	Remarks	P–P Levels *I(0)*	P–P 1^st^ Diff. *I(1)*	Remarks
**The USA**						
FDP	-1.61	-16.00∗∗∗	Order-one	-2.48	-17.09∗∗∗	Order-one
HLP	-1.60	-15.86∗∗∗	Order-one	-2.10	-17.03∗∗∗	Order-one
LCVC	-2.41	-3.08∗∗	Order-one	-4.40∗∗∗	-	Order-zero
LCVNC	-7.68∗∗∗	-	Order-zero	-6.03∗∗∗	-	Order-zero
LGSI	-3.43∗∗	-	Order-zero	-3.24∗∗	-	Order-zero
LCNHI	-5.11∗∗∗	-	Order-zero	-4.29∗∗∗	-	Order-zero
**ITALY**						
FDP	-1.24	-15.80∗∗∗	Order-one	-1.88	-16.89∗∗∗	Order-one
HLP	-1.63	-15.86∗∗∗	Order-one	-2.20	-16.91∗∗∗	Order-one
LCVC	-4.50∗∗∗	-	Order-zero	-4.46∗∗∗	-	Order-zero
LCVNC	-1.99	-22.03∗∗∗	Order-one	-2.58	-21.48∗∗∗	Order-one
LGSI	-3.25∗∗	-	Order-zero	-3.01∗∗	-	Order-zero
LCNHI	-3.19∗∗	-	Order-zero	-3.12∗∗	-	Order-zero
**FRANCE**						
FDP	-1.47	-15.84∗∗∗	Order-one	-1.95	-17.01∗∗∗	Order-one
HLP	-1.33	-15.91∗∗∗	Order-one	-2.04	-17.02∗∗∗	Order-one
LCVC	-3.59∗∗∗	-	Order-zero	-4.31∗∗∗	-	Order-zero
LCVNC	-2.51	-12.35∗∗∗	Order-one	-4.77∗∗∗	-	Order-zero
LGSI	-2.72	-11.07∗∗∗	Order-one	-2.66	-18.14∗∗∗	Order-one
LCNHI	-5.13∗∗∗	-	Order-zero	-4.89∗∗∗	-	Order-zero
**THE UK**						
FDP	-1.02	-15.79∗∗∗	Order-one	-1.62	-16.88∗∗∗	Order-one
HLP	-1.32	-15.87∗∗∗	Order-one	-2.00	-16.91∗∗∗	Order-one
LCVC	-7.40∗∗∗	-	Order-zero	-6.29∗∗∗	-	Order-zero
LCVNC	-3.97∗∗∗	-	Order-zero	-1.60	-23.06∗∗∗	Order-one
LGSI	-2.55	-3.99∗∗∗	Order-one	-2.90∗∗	-	Order-zero
LCNHI	-2.25	-9.21∗∗∗	Order-one	-2.47	-14.88∗∗∗	Order-one
**RUSIA**						
FDP	-1.07	-15.77∗∗∗	Order-one	-1.81	-16.88∗∗∗	Order-one
HLP	-1.54	-15.84∗∗∗	Order-one	-2.05	-16.90∗∗∗	Order-one
LCVC	-3.12∗∗	-	Order-zero	-3.39∗∗	-	Order-zero
LCVNC	-7.17∗∗∗	-	Order-zero	-9.60∗∗∗	-	Order-zero
LGSI	-2.86	-17.57∗∗∗	Order-one	-2.71	-18.19∗∗∗	Order-one
LCNHI	-2.99∗∗	-	Order-zero	-2.91	-18.07∗∗∗	Order-one
**TURKEY**						
FDP	-1.36	-14.41∗∗∗	Order-one	-1.50	-15.74∗∗∗	Order-one
HLP	-1.79	-14.69∗∗∗	Order-one	-2.02	-15.69∗∗∗	Order-one
LCVC	-7.53∗∗∗	-	Order-zero	-11.45∗∗∗	-	Order-zero
LCVNC	-3.49∗∗∗	-	Order-zero	-6.28∗∗∗	-	Order-zero
LGSI	-4.76∗∗∗	-	Order-zero	-4.77∗∗∗	-	Order-zero
LCNHI	-5.91∗∗∗	-	Order-zero	-5.91∗∗∗	-	Order-zero

Note: ∗∗∗ and ∗∗ signify the rejection of the null of stationarity at 1% and 5% significance levels respectively.

**Table 4:** Cointegration test for covid-19 effects on food and healthcare prices.

	The USA	Italy	France	The UK	Russia	Turkey
**Panel A: Food Prices:**						
F-Statistics	13.27∗∗∗	1.40	2.67∗∗	4.56∗∗∗	2.07∗	1.24
**Panel B: Health Prices:**						
F-Statistics	11.74∗∗	1.38	2.65∗∗	2.52∗∗	1.36	3.01∗∗

Note: The table summarizes the DARDL bounds tests. ∗∗∗, ∗∗ and ∗ indicate rejection of null hypothesis of no cointegration at 1%, 5% and 10% significant levels respectively.

The summarized results of long-run cointegration test as displayed in Table 4 indicate that a long-run relationship prevails between food prices, healthcare prices, COVID-19 cases and its induced lockdown stringency measures in all the selected worst-affected countries except for Italy (food and health prices), Russia federation (health prices) and Turkey (food prices). That is, except Italy, Russia (health prices) and Turkey (food prices), all the enlisted variables share a common trend in the long-run and therefore cointegrated.

### 4.1 The dynamic ARDL estimation

The study proceeds with the estimation of both long- and short-run relationship among the enlisted data sets in all the relevant countries. However, due to the absence of cointegration among the data sets, the long-run coefficients were not reported for Italy. Likewise, the study reported only the short-run estimates of health care and food prices for Russia and Turkey respectively. The estimated results of both long- and short-run are summarized in Table 5 (healthcare prices – Panel A) (food prices – Panel B) accordingly.

**Table 5:** Results from the Dynamic ARDL estimation (Dependent variable: Healthcare Prices).

Countries	The USA	Italy	France	The UK	Russia	Turkey
**Panel A: Dependent variable: Healthcare Prices**						
*C*	0.397∗∗∗	0.157∗∗	-0.018	0.117∗∗	0.068	0.151∗∗
*LCVC*	0.011∗∗∗	-	-0.002∗∗	-0.002∗∗	-	-0.001
*ΔLCVC*	0.065∗∗	-0.044∗∗	-0.009	-0.094∗∗∗	-0.036	-0.034
*LCVNC*	-0.007∗∗∗	-	0.001	0.001	-	-0.002
*ΔLCVNC*	-0.004	-0.005∗∗	0.001	0.001	0.001	0.002
*LGSI*	0.351∗∗∗	-	-0.030∗∗	0.012	-	0.035
*ΔLGSI*	0.066	-0.050	-0.046∗∗	0.004	-.119	0.049
*LCNHI*	-0.453∗∗∗	-	0.063∗∗	-0.012	-	-0.085
*ΔLCNHI*	-0.063	0.104	0.085∗∗∗	-0.009	-0.025	-0.170
*ECT(-1)*	-0.03∗∗	-0.02∗	-0.03∗∗	-0.03∗	-0.01∗	0.02∗
*R*^*2*^	0.52	0.61	0.72	0.45	0.34	0.38
*F-statistic*	10.21∗∗∗	2.42∗∗	1.89∗	2.66∗∗∗	2.17∗∗	2.27∗∗
*Simulations*	5000	5000	5000	5000	5000	5000
**Panel B: Dependent variable: Food Prices**						
*C*	0.421∗∗∗	0.140∗∗	-0.046	0.150∗∗∗	0.004	0.067
*LCVC*	0.011∗∗∗	-	-0.003∗∗	-0.003∗∗∗	-0.001	-
*ΔLCVC*	0.050	-0.057∗∗∗	-0.013	-0.102∗∗∗	-0.035	-0.048
*LCVNC*	-0.006∗∗∗	-	0.001	0.001∗∗	-0.003	-
*ΔLCVNC*	-0.003	-0.006∗	0.001	0.001	-1.480	0.002
*LGSI*	0.357∗∗∗	-	-0.031∗	0.015∗∗	-0.010	-
*ΔLGSI*	0.084	-0.040	-0.050∗∗	0.012	-0.122	0.025
*LCNHI*	-0.455∗∗∗	-	0.070∗∗	-0.015	0.020	-
*ΔLCNHI*	-0.085	0.095	0.097∗∗∗	-0.019	-0.027	-0.107
*ECT(-1)*	-0.33∗∗	-0.01∗	-0.02∗	-0.03∗∗∗	-0.01∗	-0.01∗
*R*^*2*^	0.52	0.62	0.53	0.84	0.15	0.06
*F-statistic*	11.11∗∗∗	2.24∗∗	1.75∗	4.25∗∗∗	2.91∗∗∗	1.19∗
*Simulations*	5000	5000	5000	5000	5000	5000

Note: ∗∗∗, ∗∗ and ∗ symbolize that the null hypothesis is rejected at 1%, 5% and 10% significant levels respectively. Panels A and B provide long- and short-run estimates for covid-19 healthcare prices nexus and covid-19 food prices nexus, respectively. ECT(-1) implies error correction term that shows the speed of adjustment to long-run equilibrium.

From the estimated results reported in Table 5 (Panels A and B), it is observed that both healthcare and food prices have significant positive autoregressive effects in all the countries except France Russia and food price in Turkey. This signifies that current prices of healthcare and food are been influenced by their past values. This outcome suggests that the task and target of stability of healthcare and food prices lies within itself. Concerning the long-run effects of COVID-19 and its induced lockdown measures on healthcare and food prices, the prevailing evidences from panels A and B demonstrate that COVID-19 cases (*LCVC*) have positive and significant effects on both prices in the USA which supports the submissions of Salisu et al., 2020a, Salisu et al., 2020b. Whereas, its effects on both prices are negative and significant in France and the UK. However, for other countries, except Italy where long-run relationship was not established, the effects of COVID-19 cases were entirely insignificant. The observed varying effects among the enlisted countries, to a greater extent reflects the quality of policy measures to cushion the effects of the COVID-19 pandemic in the country. Such measures include the health insurance policies in the US and the free national health services in the UK and some other variants in the other countries. On the other side, the effects of COVID-19 new cases *(LCVNC*) on both prices are negative and insignificant in the USA, while it is positive and significant only on food prices in the UK. This implies that new recorded cases of COVID-19 leads to increases in food prices in the UK, and price reductions in healthcare and food prices in the USA, whereas it remains neutral on both prices in all the other countries. The empirical findings of this study in terms of the effects of COVID-19 cases on healthcare and food prices in the USA contradicts the findings of Hu et al. (2021) that report negative effects of COVID-19 cases on housing prices in Australia, but it is closely related to the outcomes in France and the UK. Also, the current study provides corroborative evidence to Alam et al. (2020) which affirm that COVID-19 had significant negative impact on food and agriculture in Bangladesh. Meanwhile, the possible explanations to this varying effects, which calls for proper policy alignment suggests that in some countries, price stability measures are very strong and produces the expected outcome, while in some other countries the opposite is the case.

Considering the impacts of COVID-19 induced lockdown measures, including the government stringency index (*LGSI*) and the containment health index (*LCNHI*), the empirical evidences indicate that government stringency measure (*LGSI*) has a positive and significant impacts on healthcare and food prices in the USA and only on food prices in the UK. Meanwhile, a significant negative relationship exists between government stringency measure and healthcare and food prices in France in the long-term. For the case of containment health index *(LCNHI*), it is established that contrary to the effects of government stringency index (*GSI*), a significant negative/positive relationship exists between containment health index (*LCNHI*) and healthcare and food prices in the USA and France respectively. The above outcomes imply that the US and UK (food only) residents paid direly for healthcare services and food supplies as government stringency measures gets tougher, whereas their counterparts in France had to pay a little less for healthcare and food as the lockdown measure tightens. Conversely, containment health measure (*LCNHI*) ensured that US residents accessed healthcare services and food supplies at relatively cheaper prices as against France residents that paid more for healthcare services and food supplies. This further reflects major differences in policy directions and inclinations in the two countries which only country specific studies can elicit. For the other selected countries, including Russia and Turkey, except Italy, where long-run relationship was not established, prices of healthcare and food supplies remained unaffected despite the degree of COVID-19 induced lockdown measures. This demonstrate that the government of Russia and Turkey were able to shed their citizens from the pervasive effects of COVID-19 and its induced lockdown measures. However, it is noteworthy to mention that among the six (6) most affected countries specified herein, the Russian federation and Turkey are the least affected leading to the minimal effects of the virus and the lockdown measures on healthcare and food prices as revealed by the empirical evidences.

Furthermore, the short-term effects largely reflect the long-run effects for other countries except Italy where long-term relationship were absent. However, the prevailing empirical evidences demonstrate that both COVID-19 cases (*LCVC*) and COVID-19 new cases (*LCVNC*) had negative significant effects on both healthcare and food prices in Italy. Implying that Italian residents paid less for healthcare services and food supplies as COVID-19 cases persists within the immediate time. Also, COVID-19 induced lockdown measures (government stringency and containment health indices) did not impair residents accesses to healthcare services and food supplies within the immediate time. This suggests that within the immediate time and as the lockdown measures persists, Italian government ensured that her citizens did not suffer untold hardship in terms of access to healthcare services and food supplies. Accordingly, the F-statistics which shows the overall significance of the model demonstrates that the overall regressions are significant for the countries studied. However, the speed of adjustment (*ECT*) shows very slow adjustments to long-run equilibrium after initial short-run perturbations in all the countries especially in Italy, Russia and Turkey. Also, the adjusted (R^2^) which shows the goodness of fit between the explanatory variables and the explained variable shows high degree of predictability in most of the countries except for Russia and Turkey thereby reflecting the earlier observed effects.

For further proofs, the dynamic counterfactual simulative effects based on 5000 simulations provide graphical illustrations of the dynamics between healthcare and food prices and Covid-19 in Figures 1, 2, 3, 4, 5, 6, 7, and 8 (see Figure 9).

**Figure 1:**
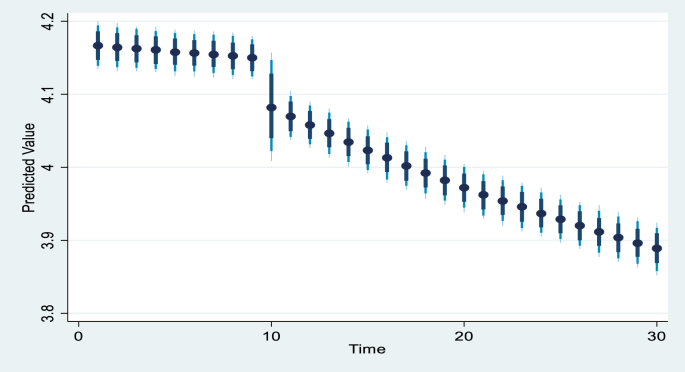
LCVC and HLP - USA.

**Figure 2:**
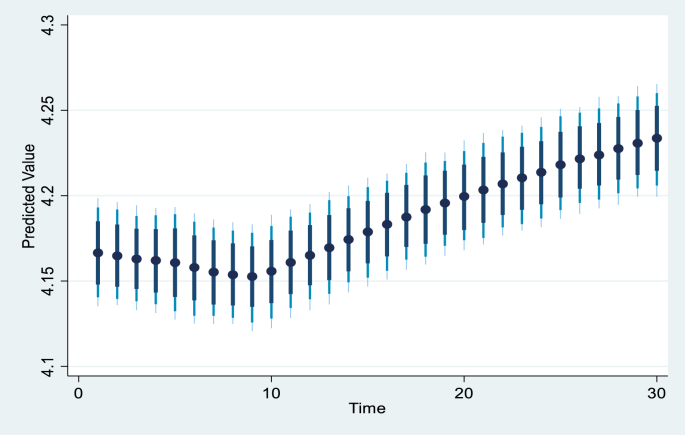
LCVNC and HLP - USA.

**Figure 3:**
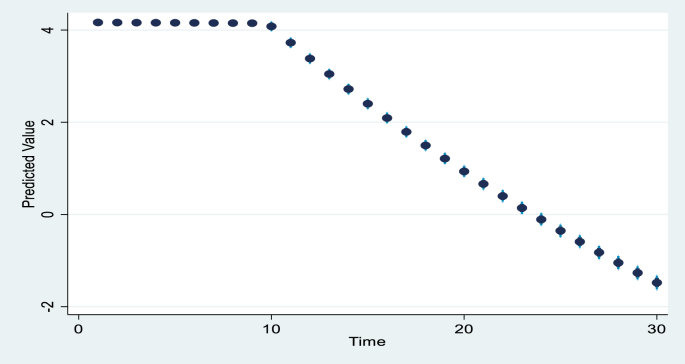
LGSI and HLP – USA.

**Figure 4:**
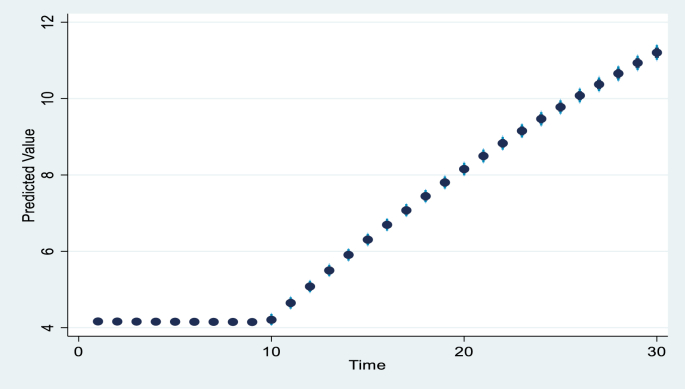
LCNHI and HLP - USA.

**Figure 5:**
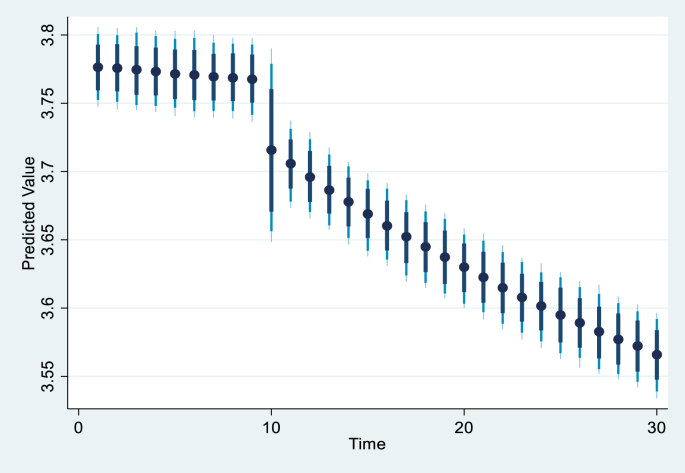
LCVC and FDP - USA.

**Figure 6:**
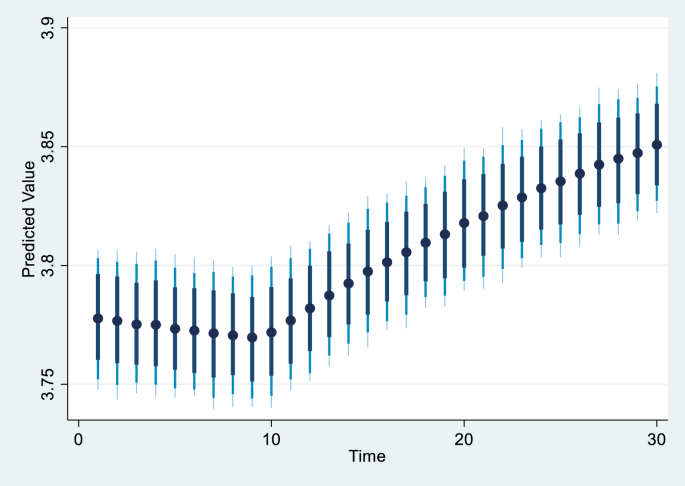
LCVNC and FDP - USA.

**Figure 7:**
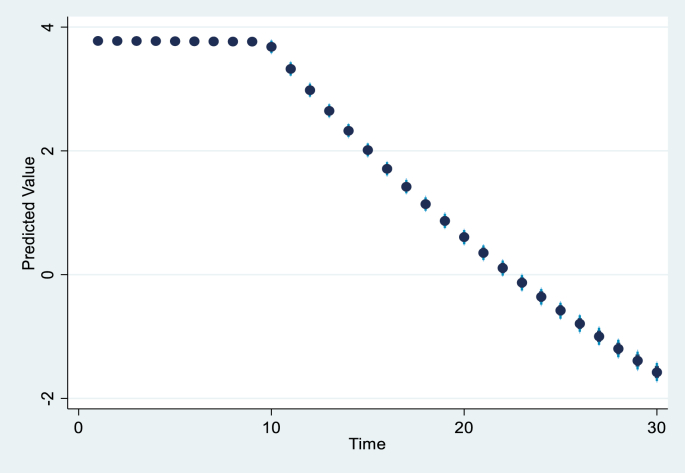
LGSI and FDP - USA.

**Figure 8:**
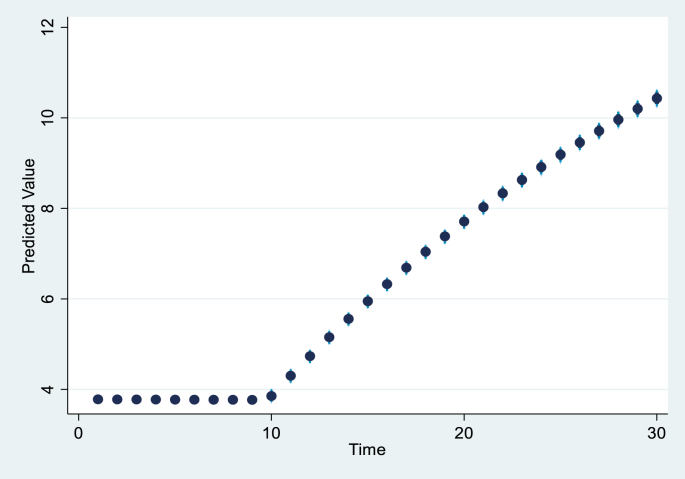
LCNHI and FDP - USA.

**Figure 9:**
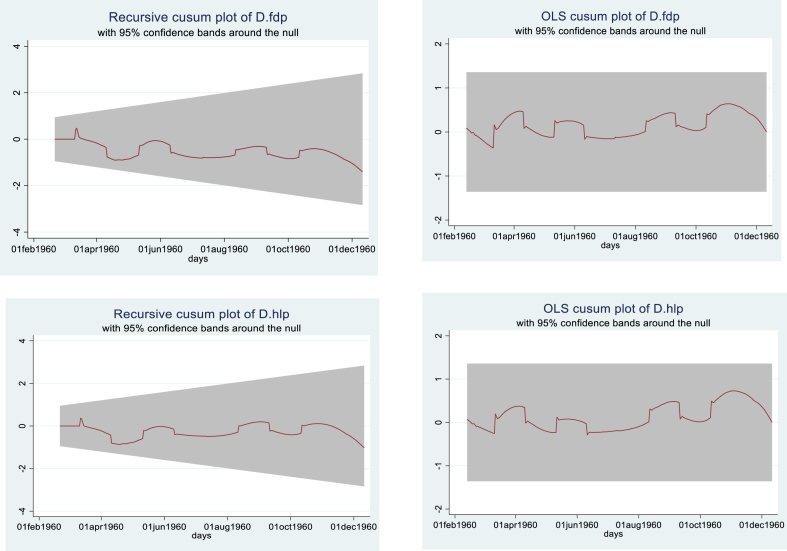
CUSUM and CUSUM^2^ graphs for models 1 and 2 – The US.

Figures 1, 2, 3, 4, 5, 6, 7, and 8 are graphical representations of the dynamic simulations of COVID-19 cases, COVID-19 new cases, government stringency index and containment health index on healthcare and food prices in the USA. Accordingly, the simulation graphs are perfect pictorial reflection of the relationship among the series. As can be clearly seen from the simulation graphs, the effects of COVID-19 cases (*LCVC*) on healthcare and food prices in the USA are clear opposite of the effects of COVID-19 new cases (*LCVNC*). Likewise, opposite effects are equally conspicuous between government stringency measure (*LGSI*) and health and containment measure (*LCNHI*) on both prices. Meanwhile, for brevity, the simulation graphs of COVID-19 and its induced lockdown measures on healthcare and food prices for other selected countries are presented only in the supplementary pages as appendix. However, due to the lack of long-run cointegration, the simulation graphs were not available for Italy. Equally not available are the simulation graphs of COVID-19 and its induced lockdown measures on healthcare and food prices in Russia and Turkey respectively due to lack of cointegration among the series.

Expectedly, the robustness and reliability of every empirical investigation, to a large extent, depends on the consequential post estimation/diagnostic tests. On this background, the empirical outcomes of the DARDL for all the enlisted countries were subjected to several post estimation enquiries. Amongst such tests are the Breusch-Godfrey and Durbin's alternative autocorrelation tests, White homoscedasticity test, and the recursive as well as OLS stability tests. Based on the outcomes of all the tests reported in Table 6, and the graphical plots of the CUSUM and CUSUM^2^ graphs, it is ascertained that the results, by all standards, are robust for policy moderations. However, for brevity, we presented the CUSUM and CUSUM^2^ graphs for the US only. The plots for other enlisted countries are available in the supplementary documents which can be made available on request.

**Table 6:** Post estimation/diagnostic tests.

Series	F-Stat (Model 1)	F-Stat (Model 2)
**The USA**		
Autocorrelation (Breusch-Godfrey LM test)	2.64 (0.10)	2.43 (0.12)
Autocorrelation (Durbin's alternative test)	2.59 (0.11)	2.38 (0.17)
Homoscedasticity (White's test)	45.56 (0.10)	38.83 (0.30)
**Italy**		
Autocorrelation (Breusch-Godfrey LM test)	3.51 (0.15)	4.62 (0.13)
Autocorrelation (Durbin's alternative test)	3.49 (0.11)	3.17 (0.12)
Homoscedasticity (White's test)	11.45 (0.24)	9.14 (0.42)
**France**		
Autocorrelation (Breusch-Godfrey LM test)	1.41 (0.23)	1.02 (0.37)
Autocorrelation (Durbin's alternative test)	1.34 (0.25)	0.97 (0.41)
Homoscedasticity (White's test)	17.37 (0.39)	34.17 (0.10)
**The UK**		
Autocorrelation (Breusch-Godfrey LM test)	3.13 (0.12)	3.74 (0.11)
Autocorrelation (Durbin's alternative test)	3.10 (0.12)	3.72 (0.12)
Homoscedasticity (White's test)	12.74 (0.13)	17.39 (0.12)
**Russian Federation**		
Autocorrelation (Breusch-Godfrey LM test)	1.42 (0.23)	1.91 (0.12)
Autocorrelation (Durbin's alternative test)	1.37 (0.24)	1.86 (0.13)
Homoscedasticity (White's test)	65.17 (0.43)	66.09 (0.40)
**Turkey**		
Autocorrelation (Breusch-Godfrey LM test)	2.37 (0.12)	2.70 (0.10)
Autocorrelation (Durbin's alternative test)	2.30 (0.13)	2.62 (0.10)
Homoscedasticity (White's test)	82.74 (0.10)	46.70 (0.74)

Note: The table summarizes the post estimation diagnostic tests. Probability values are in ().

## 5 Conclusion and policy implications

The severe impacts of the novel corona virus (COVID-19) on the global economy necessitated several studies that sought to provide relevant theoretical and empirical explanations about its links and with the notion of informing knowledge-based policy directives. Surprisingly, amid the overwhelming empirical studies, only but a few studies considered the potential link between the pandemic and food and healthcare prices. That is, most previous studies did not consider it necessary to investigate how COVID-19 and its induced lockdown stringency measures impaired access to food supply and healthcare services. On this background, the present study set for itself the task to fill this obvious lacuna in extant literature by probing the potential effects of daily COVID-19 cases and lockdown stringency measures on daily prices of food and healthcare services in the world's six worst-affected countries. In the same vein, and with the notion of providing unique empirical evidences, the study employed the newly introduced dynamic autoregressive distributed lag (DARDL) model of Jordan and Philips (2018). The DARDL model is an enhanced version of the standard ARDL model that, similar to the impulse response function (IRF) of the VAR process, provides simulative counterfactual effects of explanatory variable(s) on the explained variable.

The empirical evidences from the analysis demonstrate that prices of healthcare services and food items follow an autoregressive function. Implying that their current prices even during the pandemic are being influenced by their prices in the past. Such being the case, to shield the citizens from the severe effects of COVID-19 case and its induced lockdown measures, efforts should be made to contain and control such spillover effects arising from past prices mostly during the pandemic. Equally noted is that COVID-19 cases affected consumers access to healthcare and food supplies in the USA in terms of rising prices, whereas, it is the new recorded cases that affected citizens access to food supply in the UK. This requires that the US and the UK government pay more attention to COVID-19 cases and COVID-19 new cases respectively to alleviate the sufferings of her citizens. However, for the other selected countries, both prices remained unaffected despite the magnitudes of COVID-19 cases and new cases. Meanwhile, the government of these countries need to keep a close watch on these prices to ensure that they remain unaffected and stable even amid the second wave of the pandemic and beyond. Furthermore, the study discovered that government stringency index (*LGSI*) restricted residents access to healthcare services and food supplies in the US and food supplies in the UK, whereas, it was the containment health index that hampered consumers access to healthcare services and food supplies in France. Accordingly, to reduce the severe implications of the lockdown measures on the citizenry in terms of rising prices of healthcare services and food supply, the US and the UK government should pay more attention and reconsider her stringency measures (*LGSI*), while the government of France has to reconsider her stance on containment and health measures. Conclusively, the study, to a large extent, suggests the acceptance of the hypothesis that COVID-19 cases and its induced stringency measures leads to rising food and healthcare prices in the enlisted countries with minor variations in some countries as pointed out earlier.

Unarguably, most studies that rely on time series mostly from an evolving phenomenon like the COVID-19 for empirical modeling might suffer notable limitations and the present study may not be an exception. As such, the inferences thereof may not be all encompassing. Meanwhile, such notable limitations do not in any way affect the integrity and findings of the present exposition. More so, empirical details on the focal point of this study (COVID-19) are still evolving. Therefore, more studies with similar orientation are required to confirm the findings of the present study using the adopted modelling technique (DARDL) or other relevant procedures. Furthermore, the inclusions of other related macroeconomic variables like the exchange rate, inflation as so on, and the enlistment of other affected countries could elicit better understandings of the effects of the pandemic on the global economic landscape leading to wider ameliorative policy options.

## Declarations

### Author contribution statement

Emmanuel Uche: Conceived and designed the experiments; Performed the experiments; Analyzed and interpreted the data; Wrote the paper.

Samuel Nnamdi Marcus: Conceid designed the experiments; Analyzed and interpreted the data; Wrote the paper.

Lionel Effiom: Analyzed and interpreted the data; Wrote the paper.

Chijioke Okoronkwo: Performed the experiments; Analyzed and interpreted the data; Wrote the paper.

### Funding statement

This research did not receive any specific grant from funding agencies in the public, commercial, or not-for-profit sectors.

### Data availability statement

Data associated with this study has been deposited at Our world in data.

### Declaration of interests statement

The authors declare no conflict of interest.

### Additional information

No additional information is available for this paper.

## Appendix A Supplementary data

Appendix - Covid -and food prices Appendix - Covid -and food prices

## Footnotes

1 https://ourworldindata.org/coronavirus-data?country.

2 https://ourworldindata.org/coronavirus-data?country.

3 https://ourworldindata.org/coronavirus-data?country.
